# Development of a decision support intervention for family members of adults who lack capacity to consent to trials

**DOI:** 10.1186/s12911-021-01390-4

**Published:** 2021-01-28

**Authors:** Victoria Shepherd, Fiona Wood, Richard Griffith, Mark Sheehan, Kerenza Hood

**Affiliations:** 1 Centre for Trials Research, Cardiff University, Neuadd Meirionnydd, Heath Park, Cardiff, CF14 4YS UK; 2 PRIME Centre Wales, Division of Population Medicine, Cardiff University, Heath Park, Cardiff, CF14 4YS UK; 3 College of Human and Health Studies, Swansea University, Singleton Park, Swansea, SA2 8PP UK; 4 Ethox Centre, University of Oxford, Big Data Institute, Old Road Campus, Oxford, OX3 7LF UK

**Keywords:** Decision aid, Research, Surrogate decision maker

## Abstract

**Background:**

Informed consent is required for participation in clinical trials, however trials involving adults who lack capacity to consent require different enrolment processes. A family member usually acts as a proxy to make a decision based on the patient’s ‘presumed will’, but these decisions can be challenging and families may experience an emotional and decisional burden. Decisions made on behalf of others are conceptually different from those made for ourselves. Innovations have been developed to improve informed consent processes for research, including a number of decision aids, however there are no interventions for proxies who are faced with more complex decisions. This article outlines the development of a novel decision aid to support families making decisions about research participation on behalf of an adult who lacks capacity to consent.

**Methods:**

Decision support interventions should be developed using rigorous and evidence-based methods. This intervention was developed using MRC guidance for the development of complex interventions, and a conceptual framework for the development and evaluation of decision aids for people considering taking part in a clinical trial. The intervention was informed by a systematic review and analysis of existing information provision. Previous qualitative research with families who acted as proxies enabled the development of a theoretical framework to underpin the intervention. The intervention was iteratively developed with the involvement of lay advisors and relevant stakeholders.

**Results:**

Previous research, theoretical frameworks, and decision aid development frameworks were used to identify and develop the intervention components. The decision aid includes information about the proxy’s role and utilises a values clarification exercise and decision support methods to enable a more informed and better-quality decision. Stakeholders, including those representing implementers and receivers of the intervention, contributed to the design and comprehensibility of the decision aid to ensure that it would be acceptable for use.

**Conclusions:**

Frameworks for the development of decision aids for people considering participating in a clinical trial can be used to develop interventions for family members acting as proxy decision-makers. The decision support tool is acceptable to users. Feasibility testing and outcome measure development is required prior to any evaluation of its effectiveness.

## Background

Informed consent is considered to be the cornerstone of ethically conducted medical research [1, 2]. However, there are times when patients are unable to provide consent for themselves due to a cognitive impairment, which may be associated with a neurodegenerative condition such as dementia or resulting from an acute medical emergency or accident. Adults considered to be unable to make a particular decision for themselves at the time the decision or action needs to be taken are described as lacking decision-making capacity (Part 1 (2(1) and (2)) [3]. In England and Wales an assessment process laid down in the Mental Capacity Act 2005 is used to determine whether someone has capacity to make a particular decision [3]. Where a potential research participant lacks capacity to consent, someone close to the patient, usually a family member, is approached to make a decision about research participation on their behalf.

Arrangements governing who is legally authorised to decide about enrolment in research differ across legal jurisdictions and may also vary according to the type of research. In England and Wales, the Mental Capacity Act 2005 (MCA) governs most types of research and has provision for consulting someone who knows the person with impaired capacity well to act as consultee and advise about research participation on their behalf [3]. The family member or friend acting as consultee is provided with information about the project and asked what the patient’s likely wishes and feelings would be about taking part in the project if he or she had capacity [3]. However, responsibility for deciding whether to include the patient lies ultimately with the researcher [3]. For clinical trials of investigational medicinal products (CTIMPs), the Medicines for Human Use (Clinical Trials) Regulations 2004 (CTR) applies across the UK [4]. Under the CTR, the family member or friend is termed a legal representative and provides informed consent on behalf of the patient based on what they would have wanted had they the capacity to choose for themselves, their ‘presumed will’ [4]. The term proxy is used in this paper to include both consultees and legal representatives.

Previous research has shown that whilst families are supportive of being involved in proxy decisions about research [5], it can be a difficult task [6]. Family members are responsible for making a decision that may potentially have far-reaching consequences for the health and welfare of another person. Proxies are acutely aware of the moral difference between deciding for themselves and deciding for others [7], and may experience an emotional and decisional burden as a result. A recent systematic review of proxy decision-making for research found that the weight of making a decision on behalf of another person, together with the uncertainty about how decisions ought to be made by proxies, is burdensome for proxies [8]. One study reported that nearly all proxies experience some degree of burden when making decisions about research [6]. Our recent qualitative study found that making decisions about research was problematic for some proxies who were concerned about making what they considered to be the ‘right’ decision for the person they represented [9]. The study found that some proxies may benefit from decision support in order to make an informed decision about research participation [9].

### Decision support interventions for research participation decisions

Decision support interventions, also known as decision aids (DAs) are interventions that are increasingly being used to support patients who are making choices about their healthcare by making their decisions explicit, and providing information about their options and the associated benefits and harms [10]. DAs differ from traditional information materials in many ways, including that they are not narrowly focused on improving the delivery of information [11] nor intended to encourage a particular choice or action [12] but are intended to facilitate patient involvement in decisions, leading to decisions which are informed and consistent with their own values [13, 14]. DAs can take many forms, for example a paper-based booklet, video, or web-based tool [15]. More recently, decision aids have been developed for decisions about participation in trials [16], however proxy decisions about research have not previously been considered a target for improvement.

In health care, patients’ decisions about treatment and screening are often described as ‘preference sensitive’, where the relative importance a patient attaches to various outcomes and processes has a large, if not determining, influence on what is decided [17]. Quality decision-making is therefore said to be the extent to which the chosen option matches the informed decision-maker’s values for benefits, harms, and uncertainties [18]. The effectiveness of DAs on improving decisions (making them ‘good’ decisions) can be viewed as influencing two constructs: (i) the quality of the decision-making process and (ii) the quality of the choice that is made (i.e. decision quality) [19]. There is growing evidence of the effectiveness of DAs in improving both the quality of the decision-making process and decision quality for healthcare decisions [19]. A recent Cochrane review established that decision aids can improve knowledge, reduce decisional conflict, clarify expectations of possible benefits and harms, lead to choices consistent with informed values, and result in greater participation in decision making [10].

A small number of interventions have been developed to promote informed decision-making about participation in clinical trials [20–22]. Of the small number of DAs that aim to improve decision-making for trial participation (rather than aiming to solely improve the presentation of information or mode of delivery) most focus on specific oncology trials [11]. These DAs show some potential promise in improving key decision outcomes such as knowledge, values clarification, and decision conflict, while not negatively impacting recruitment or intention to participate [20, 21, 23]. A recent systematic review of decision aids for trials only identified one study that evaluated the effectiveness of decision aids compared to standard information in the informed consent process for clinical trials [16]. The reviewers concluded that more high quality randomised controlled trials of decision aids to support the informed consent process for clinical trials are needed [16]. In non-oncology conditions, a qualitative exploration of stakeholders’ perceptions of decision aids for randomised controlled trials that are currently in development suggested that decision aids have the potential to better engage potential participants in the decision-making process and allow them to make more personally relevant decisions about their participation [24].

### Need for decision support interventions for proxy decisions about research participation

Despite these innovations to improve informed consent decisions, and proxy decisions about healthcare (e.g. for people living with dementia [25]) there are no interventions for proxies making decisions about research participation which, it could be argued, are more complex decisions. Legal and ethical frameworks require proxy decisions about research participation to be based on what the wishes and feelings of the person lacking capacity to consent would be about taking part in the study [3] or, for a clinical trial, representing their ‘presumed will’ [4]. This requires the proxy to first determine what the wishes and preferences of the person they are representing would be about participating, and then make a decision based on this determination. Proxy decisions can therefore also be viewed as preference sensitive decisions, but they are based on the patient’s preferences and values rather than their own. Whilst some patients may have discussed their preferences about participating in any research in the event of a loss of capacity to consent, in practice very few will have done so [8], and so making a decision that represents their wishes may be challenging for proxies. Interventions to reduce the burden and decisional conflict experienced by proxies are needed in order to support family members through what can be an emotional and challenging decision [9], and so may help address the current exclusion of adults who lack capacity from research [26].

A DA for proxy decision-makers could improve the decision-making process through helping the proxy determine what the person’s preferences would be, and decision quality by ensuring that the choice made matches those preferences. As there is a lack of a DA for proxy decisions about research, and DAs are decision‐specific interventions that are unlikely to produce a benefit in other decision contexts [27], development of a decision-specific intervention is needed. This paper describes the development of a novel intervention to support decision-making by family members when making decisions about research participation on behalf of a patient who lacks capacity to consent.

## Methods

### Development of the decision support intervention

**Fig. 1:**
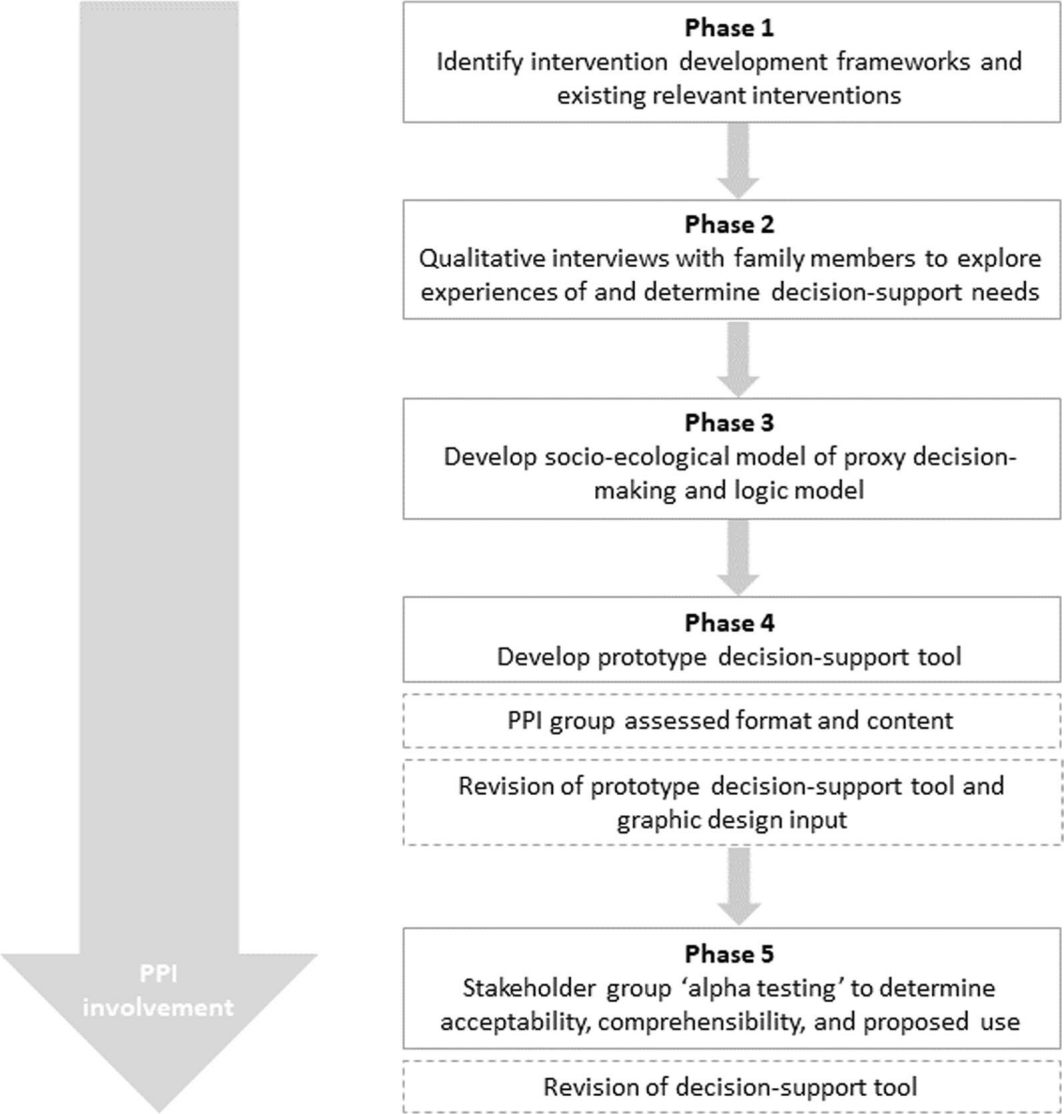
Intervention development process flow diagram

As the interest in DAs for trial participation grows, so does the importance that their development is both rigorous and evidence-based [11]. The development of this DA for proxy decision-making was informed by a recently proposed framework for the development and evaluation of decision aids for people considering taking part in a clinical trial [11]. The framework proposed by Gillies and Campbell consists of five steps: (1) selecting an underpinning theoretical approach to the development process; (2) developing the decision aid; (3) assessing and testing feasibility; (4) evaluating the decision aid; and (5) implementing the decision aid in practice [11]. The first two steps of the framework are reported in this paper and an overview of the process is outlined in Fig. 1.

## Results

### Phase 1: identify relevant intervention development frameworks

The initial phase of the development process was to identify appropriate frameworks that inform the development of complex interventions in general, and then those specifically for decision aid interventions from both a theoretical and practical perspective.

### Intervention development frameworks

The Medical Research Council (MRC) framework for developing and evaluating complex interventions is widely used [28]. It describes an iterative process of development, feasibility/piloting, evaluation, and implementation of interventions [28], however, it has limited detailed guidance for the development phase itself. Recently, a model which focuses on the intervention development phase (6Squid) has been developed which describes how the process can be broken down into six steps, although in practice the process is non-linear and collaborative [29]. Researchers have proposed combining the elements of the development phase of the MRC Framework with elements of the 6Squid model to enhance the intervention development through a more comprehensive approach. This ‘enriching’ approach to the development phase [30] has been used for the development this decision support intervention. The methods used in the 6Squid steps are shown in Table 1.

**Table 1:** Six steps in development of the decision aid intervention

Step in intervention development	Method used
(1) Define and understand the problem and its causes	Systematic review, content analysis, qualitative data
(2) Clarify which causal or contextual factors are malleable	Logic model
(3) Identify the change mechanism	Qualitative data
(4) Identify how to deliver the change mechanism	Qualitative data, review of current DAs
(5) Test and refine on small scale	Review by PPI and stakeholder groups
(6) Collect sufficient evidence of effectiveness to justify rigorous evaluation/implementation	Feasibility study^a^

^a^Feasibility study to be conducted as part of a follow-on project

### Decision aid development frameworks

The Ottawa Decisional Support Framework (ODSF), which is an evidence-based, practical, mid-range theory for guiding patients making health or social decisions, was used to develop the DA [18]. This theoretical framework consists of three components: decisional needs; decisional support; and decisional quality. The framework for the development and evaluation of decision aids for people considering taking part in a clinical trial outlined by Gillies and Campbell identified the ODSF as being a highly relevant theoretical framework to use when developing decision aids for trial participation [11]. This is because the ODSF asserts that decisional needs affect decisional quality, such as making informed and values-based choices, which in turn affect action, behaviour, and emotions such as regret [18]. These concepts are considered by Gillies and Campbell to map well onto the issues within trial participation decisions [11] which are known to be preference-sensitive decisions [31] as is also the case for proxy decisions.

In addition to ODSF, the International Patient Decision Aids Standards (IPDAS) framework of quality criteria for patient decision aids was used to guide the practical development of the decision aid [32]. This evidence-informed framework provides a set of criteria which aims to improve decision aid content, development, implementation, and evaluation [32]. Alongside the IPDAS framework, a 44-item minimum standards ‘checklist’ version has been developed that is designed to rate the quality of the development process and decision-making design elements [33]. Relevant items from the IPDAS Minimal criteria v4.0 were used to inform the development of this decision support intervention. Checklist items considered not relevant, and hence excluded from the development process, included: information relating to the condition or problem and associated probabilities as this would be included in the Participant Information Sheet provided about the study (and so would be used alongside the DA) and information related to screening or tests as this was not applicable.

IPDAS guidelines for the development of patient DAs recommend that they include a process for helping people clarify their values [32]. These processes, usually termed values clarification methods (VCM), are defined as strategies intended to help individuals to evaluate the desirability of options or attributes of options within a specific decision context, in order to identify which option they prefer [34]. VCMs can be implicit and non-interactive (e.g., the individual thinks about what is important to their decision), or explicit and interactive (e.g., using a rating scale for each attribute to reflect the importance of each to their decision) which is much more widely studied [34]. Whilst DAs have been found to be effective in reducing decisional conflict and increasing knowledge, the effect of specific strategies such as VCMs is less clear [35]. VCMs encourage patients to follow deliberative and analytical processes when comparing available choice options, although deliberation may not always be beneficial as it may overshadow important intuitive feelings that are more difficult to formulate but may be just as important in decision making [36]. However, the use of VCMs in DAs decision aids is now widespread, with 57.1% of DAs in a recent systematic review including explicit methods to clarify values [10] and has therefore been included in this DA (further information is provided in the next section).

### Review of existing decision aids for proxies

Although the search strategy for the systematic review of decision aids for trials specifically sought to include studies that included guardians of, or proxy decision makers for, potential trial participants, no existing decision aids for use by proxies have been identified [16]. However, a number of tools have been developed for proxies who are making other decisions on behalf of a person who lacks capacity, such as around the use of antipsychotic medication, place of care, retiring from driving due to dementia, and receiving mechanical ventilation [15, 37–39]. The DAs intended for use by proxies invariably make the assumption that the evidence that decision tools are effective in increasing knowledge and reducing decisional conflict is transferable to proxy decision-making, and there is some limited evidence of this. A small study examining the impact of a DA on proxy decision-makers’ perceptions of feeding options for people with dementia found it improved knowledge scores and reduced conflict [40]. A feasibility study of an intervention to support family carers making decisions about place of care for a person living with dementia also found that it reduced decisional conflict, although it did not remove all barriers to decision-making and some unresolved conflict remained [37]. However, there is need for further trials to fully establish their effectiveness [25].

### Phase 2: prior research which informed the intervention development

Clarifying the problem using the existing research evidence and in consultation with stakeholders is considered to be the first step in intervention development [29]. Phase 2 built on a systematic review which was conducted to synthesise the existing empirical evidence [8] which, together with a content analysis of information already provided to proxies [41], indicated that proxies are generally well provided with information about the study itself, but are not well informed about their role as proxy decision-maker. Whilst there is a paucity of literature regarding decision support for proxy decisions about research, the relationship between the parties involved appears to determine the kind of decision-making process used and impacts on the way decisions are achieved [42]. This decision support intervention therefore focuses on close family members acting as proxies, rather than others who can also legally act as proxy decision-makers for research such as members of the healthcare team [43].

We conducted a qualitative study with family members acting as proxies which showed that they use the patient’s expressed wishes about research where these are available and, where these were not known, use their in-depth knowledge of the person’s values and preferences to facilitate decision-making on their behalf [9]. However, other proxies described it as being a difficult and challenging decision. Improving the decision-making process was recognised as being much more than just ensuring the proxy had received adequate information. Proxies thought that greater decision support when considering research decisions would help in the future, which included orientating them towards considering the person’s own views and preferences [9]. Proxies suggested that this support could take the form of a different sort of information sheet which covered their role as proxy decision-maker, and that the DA should include items that they considered would support proxies when making decisions about research (Table 2).

**Table 2:** Items for inclusion in decision aid from the qualitative interview findings

1. Why people with cognitive impairment are included in research
2. That the proxy’s decision or advice should be based on what the person’s wishes and feelings about taking part would be if they had capacity to decide
3. That the proxy should consider if there is any reason why the person would not have wanted to participate
4. The relevant advantages and disadvantages and how they relate to the person themselves
5. That the person should be involved in the decision as much as possible
6. That the proxy can take time to decide and they can always change their mind

As the majority of the participants were proxies of someone living with dementia, the intervention was developed with this population as its focus, although there is no reason to suggest it would not be appropriate for proxies of people with other conditions associated with cognitive impairment.

### Collaborative development with stakeholders

Consultation with relevant stakeholders is an important step in the intervention development process [29]. This intervention was part of a larger project which benefitted from having the support of a lay advisory panel from the initial conception of the project through to the development of the intervention itself. The lay advisory group, also known as a Public and Patient Involvement (PPI) group attended a discussion meeting to review the first draft of the decision aid, broadly following a cognitive debriefing approach to instrument development which can identify difficult or confusing areas of the item being reviewed and propose a better version [44]. The aim is also to identify whether the interpretation of an item differed between those reviewing it [44].

Additionally, a larger group of stakeholders was convened and consulted who had experience as either a researcher who involves people who lack capacity in their research, family member who may be approached to act as a proxy, or those familiar with supporting family members of people with dementia. The importance of the involvement of practitioners and other stakeholders in developing and prototyping interventions, to ensure that they can be adopted, implemented and maintained in the contexts for which they are intended, is emphasised in many of the frameworks guiding intervention development [29, 45]. Their role is important throughout the process, but particularly when determining the content, format, and delivery of the intervention [29]. Acceptability of an intervention has been defined as ‘a multi-faceted construct that reflects the extent to which people delivering or receiving a healthcare intervention consider it to be appropriate, based on anticipated or experienced cognitive and emotional responses to the intervention’ [46], and can be considered to have both cognitive and affective components [47]. This stage forms the ‘alpha’ testing of the intervention which is described as an iterative process of testing by people directly involved in the development process, and considered to be a necessary part of the development of decision aids [48].

### Phase 3: development of a socio-ecological model of proxy decision-making

The term ‘complex interventions’ are primarily used to refer to interventions as system changes that are focused on health promotion, social interventions, and public health more broadly. Complexity is increasingly being conceived in terms of how interventions interact with their contexts, where contexts are broadly defined as any feature of the circumstances in which an intervention is conceived, developed, implemented and evaluated [45]. There is a call for intervention researchers to move away from viewing interventions as discrete bundles of components which can be described in isolation from their contexts, and better understand the systems into which change is being introduced [49]. Thus there is a focus on interventions viewed as disruptions to complex systems, rather than on the intrinsic properties of interventions [50]. Decision aids, particularly this one to be used by proxies, can be considered to have properties of complexity. It is a complex intervention with several interacting components [51], entailing complex behaviours and a range of effects [52], and also as an event occurring within inevitably complex systems such as within a family, legal and ethical frameworks, and a healthcare system.

**Fig. 2:**
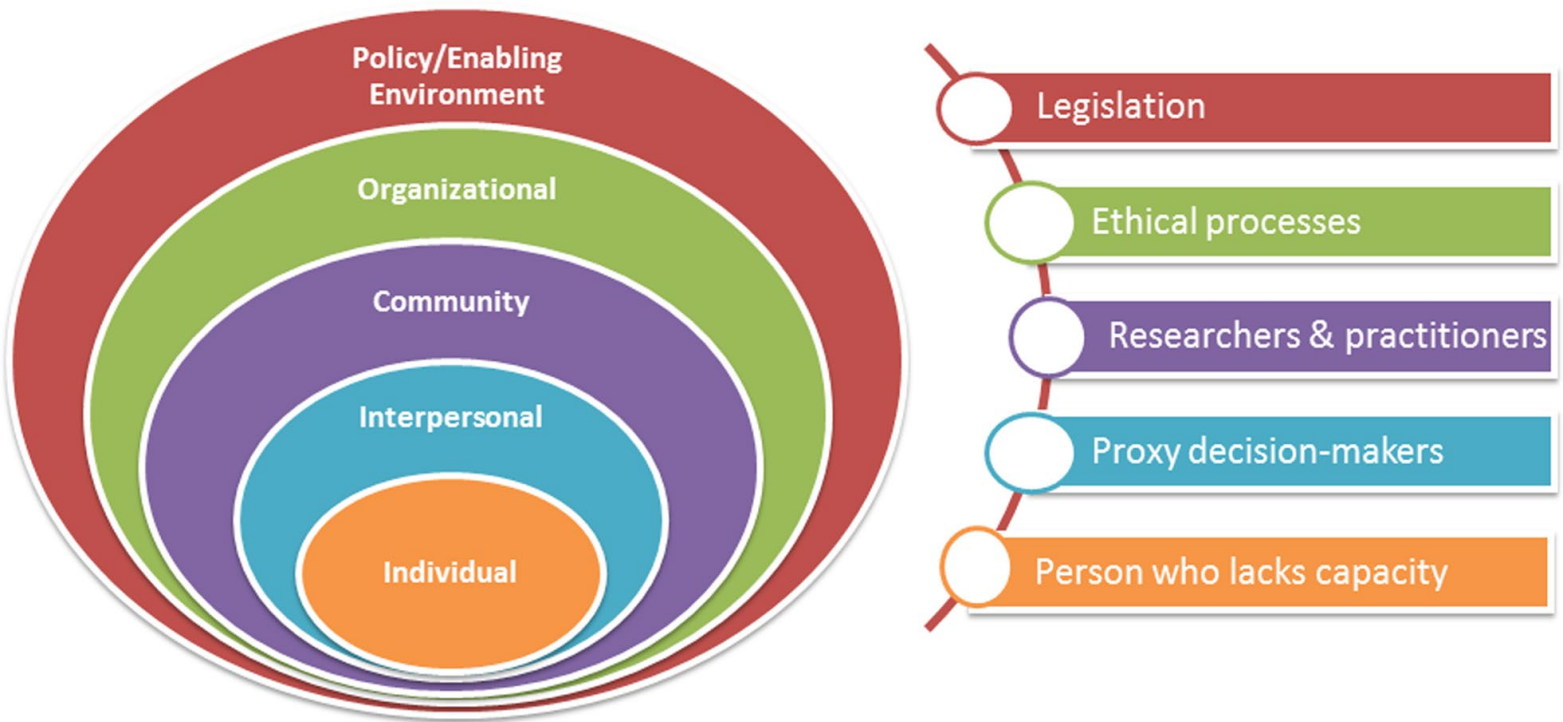
Socio-ecological model of proxy decision-making for research

Proxy decision-making for research, whilst not traditionally thought of as a behaviour, is similarly situated within complex systems which can be perceived as having a number of levels which have contextually dependent dynamic interactions between them. Developing a socio-ecological model (SEM) as phase 3 of the process was found to be a useful organising construct for proxy decision-making. It was adapted from the model most commonly used for health promotion and behaviour change interventions [53]. An ecological perspective recognizes that individuals are located within a broader social context [54, 55]. In this model, behaviour is conceived as being determined by five levels of analysis: intrapersonal factors, interpersonal processes, community factors, institutional/organisational factors, and public policy or socio-cultural factors [56]. Developing the SEM enabled an exploration of the many different factors or levels involved, including the legal frameworks at both a policy and socio-legal level, the ethical governance processes involved, the knowledge and attitudes of the researcher and practitioner community, the interpersonal relationship between the proxy and the person they represent, which ultimately centre around the person who lacks capacity to consent. The main influences on the issue being examined were classified according to the socio-ecological model distinguishing between the different levels of the model (Fig. 2).

In order to clarify which causal or contextual factors might have the greatest scope for change, a logic model [29] was iteratively developed (Additional file 1: Logic model for proxy decision support intervention). Logic models are an important tool for implementing a theory‐based approach [57]. Logic models can help in areas of complexity by depicting intervention components and the relationships between them and displaying interactions between the intervention and the system within which it is to be implemented [58].

### Phase 4: develop prototype decision-support intervention

**Fig. 3:**
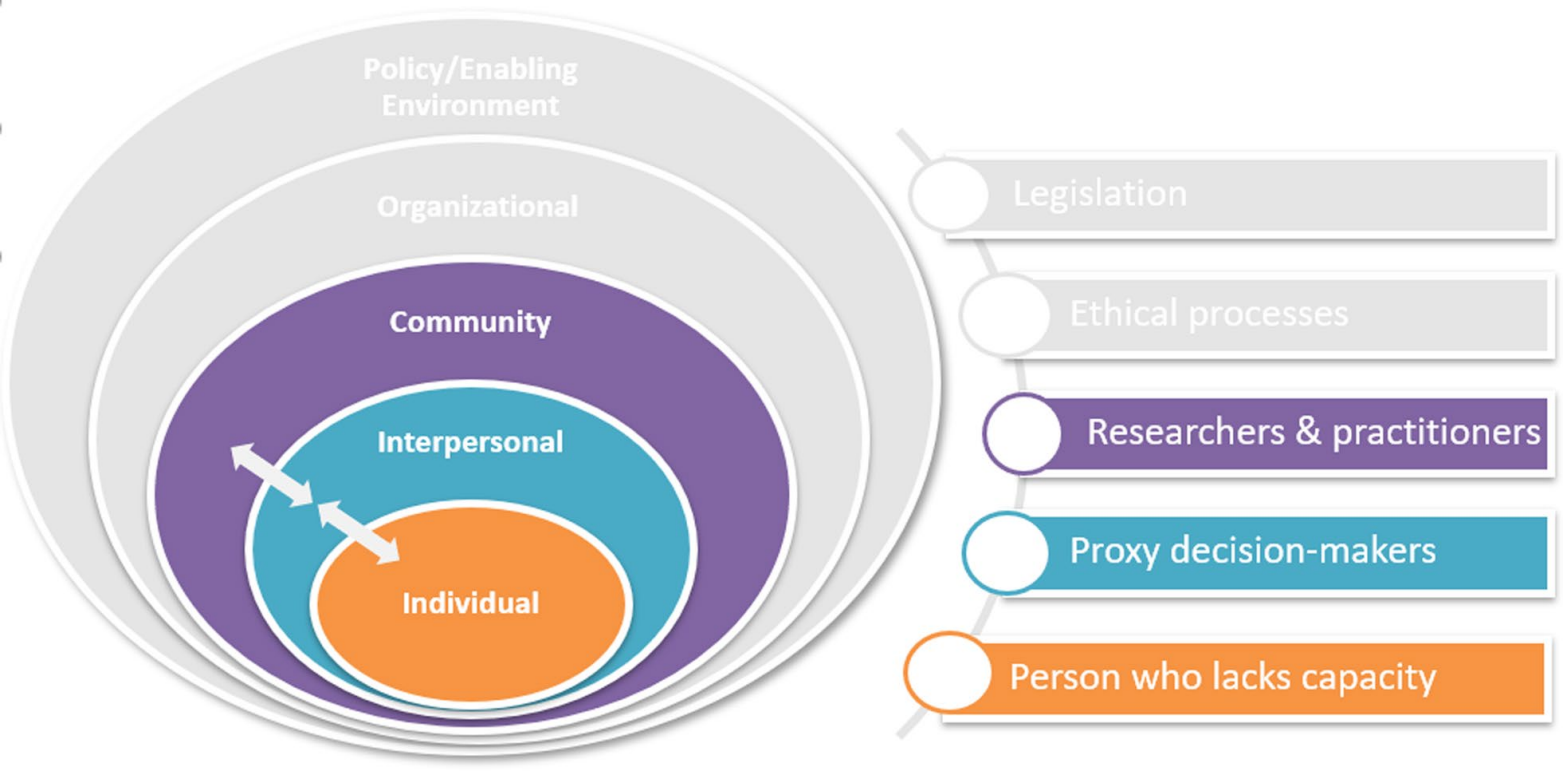
Location of intervention to support proxy decision-making for research in socio-ecological model

A decision aid was developed for proxy decision-makers that would supplement the study-specific information contained in the Participant Information Sheet (PIS) and be delivered within a consultation with the researcher or clinician providing information about the research study or clinical trial. In addition to the need for decision support for proxies arising from the qualitative interviews, the previous research indicated that there were additional researcher/clinician informational needs that needed to be addressed. For the purposes of this intervention, it suggested that an additional education and training component was required to provide information about the legal frameworks to those who would be delivering the intervention. A short one-page guide to the key messages of the decision support booklet was also developed as part of the intervention. The intervention (the DA with an additional education component for the clinician/researcher undertaking recruitment) spans the individual-interpersonal-community spheres of the SEM of proxy decision-making. The aim of the intervention was to improve informed decision-making by proxies that better reflects the wishes and preferences of the person they represent, whilst ensuring it pays attention to the ethical principles and corresponds to the relevant legal frameworks (Fig. 3). The intervention also needed to be attentive to the informational needs of the healthcare professional, social care practitioner, or researcher who will be delivering the intervention to ensure that they are knowledgeable and confident in the inclusion of adults lacking capacity in research.

### Format and content of the decision aid

**Fig. 4:**
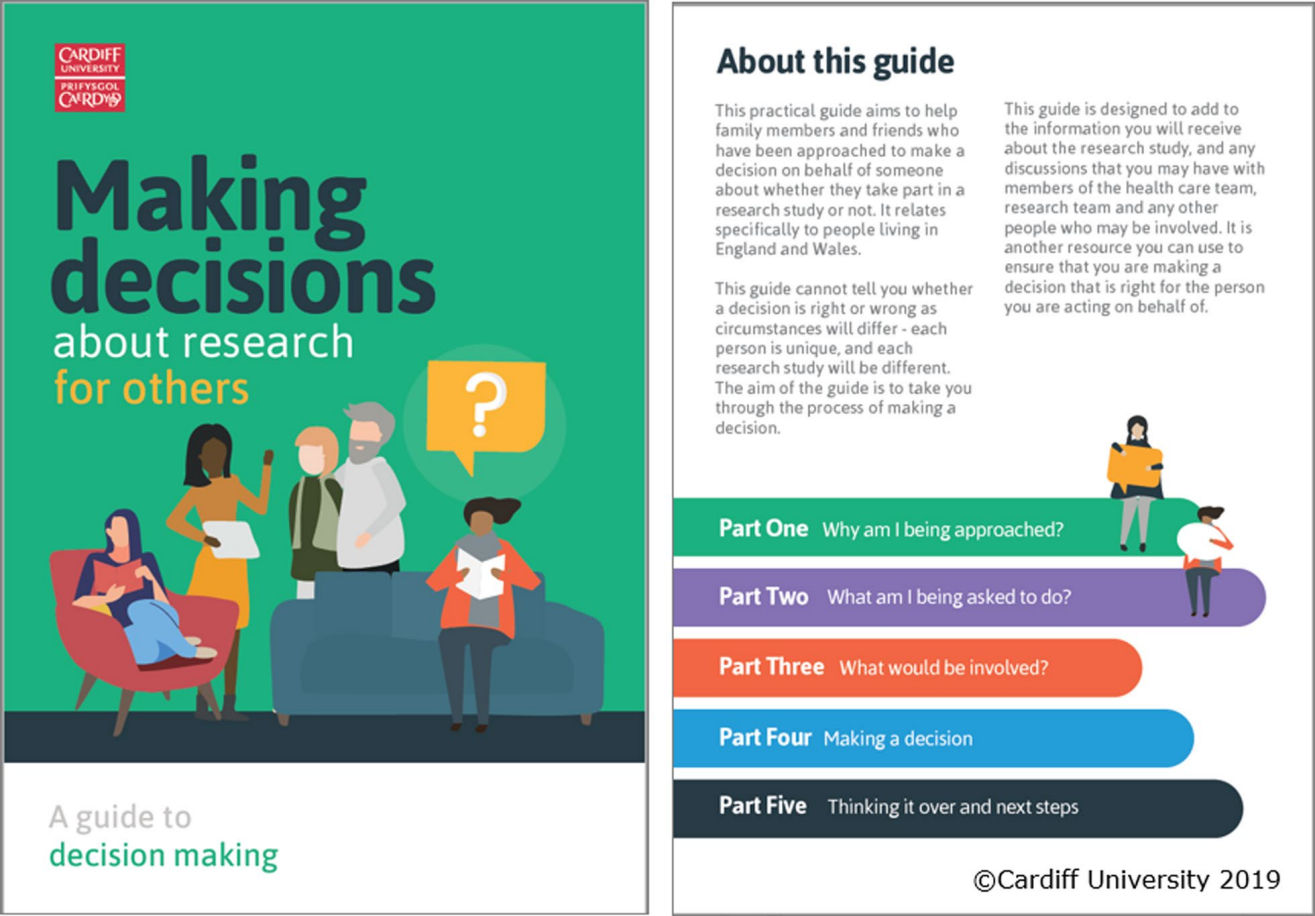
Cover page and content list of the decision aid for family members making decisions about research

A prototype of the DA was developed in phase 4 and iteratively refined following consultation with the lay advisory and stakeholder groups. The DA was presented as a 12-page A5 paper booklet which could be printed or read as a PDF document with ‘interactive’ features that encouraged proxies to identify the advantages and disadvantages of the research study being considered (Fig. 4).

The prototype DA was enhanced by a graphic designer to improve the accessibility and visual impact of the tool. A range of strategies were used to enhance reader understanding of the DA. Information was presented clearly and concisely using colour-coded sections to navigate the booklet. Readability scores [59] were assessed which showed that a Flesch Reading Ease score of 69.9 (fairly easy to read) was achieved and a Flesch-Kincaid Grade Level suggested that most 8th grade students (age 13–14 years old) would be capable of reading the booklet. Space, with prompts, was provided for notes and questions at various points in the booklet, and a space for additional questions was provided at the end of the booklet.

**Fig. 5:**
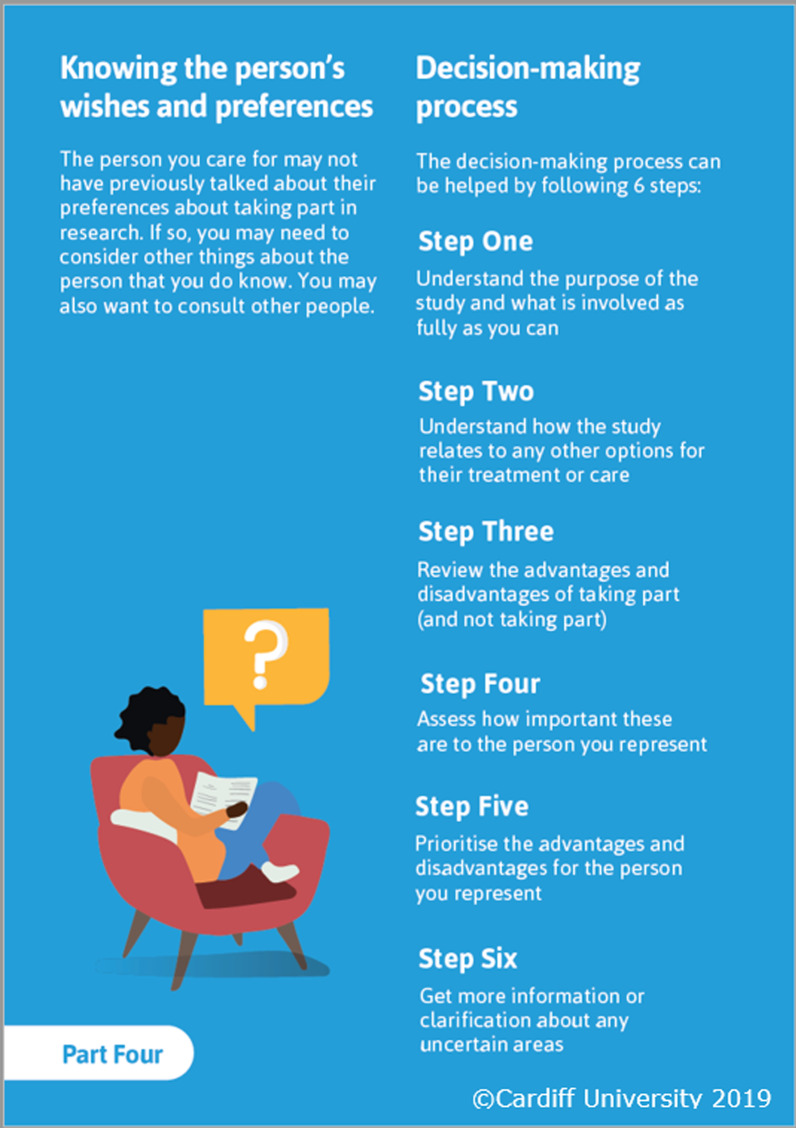
Six stage decision-making process detailed in the decision aid

The content was presented in the order used in other examples of DAs for decisions about clinical trials [20, 24]. This presented information about their role (including clarifying whether they were being asked to provide advice or consent), understanding any advantages and disadvantages of participation, followed by structured guidance in deliberation or ‘making a decision’ that was adapted from existing clinical trial DAs [20, 24]. The suggested decision-making process was through the six steps detailed in Fig. 5. adapted from Juraskova et al. [60]. This widely used DA component was included in order to provide some structure to the decision-making process that proxies felt they would benefit from [9].

**Fig. 6:**
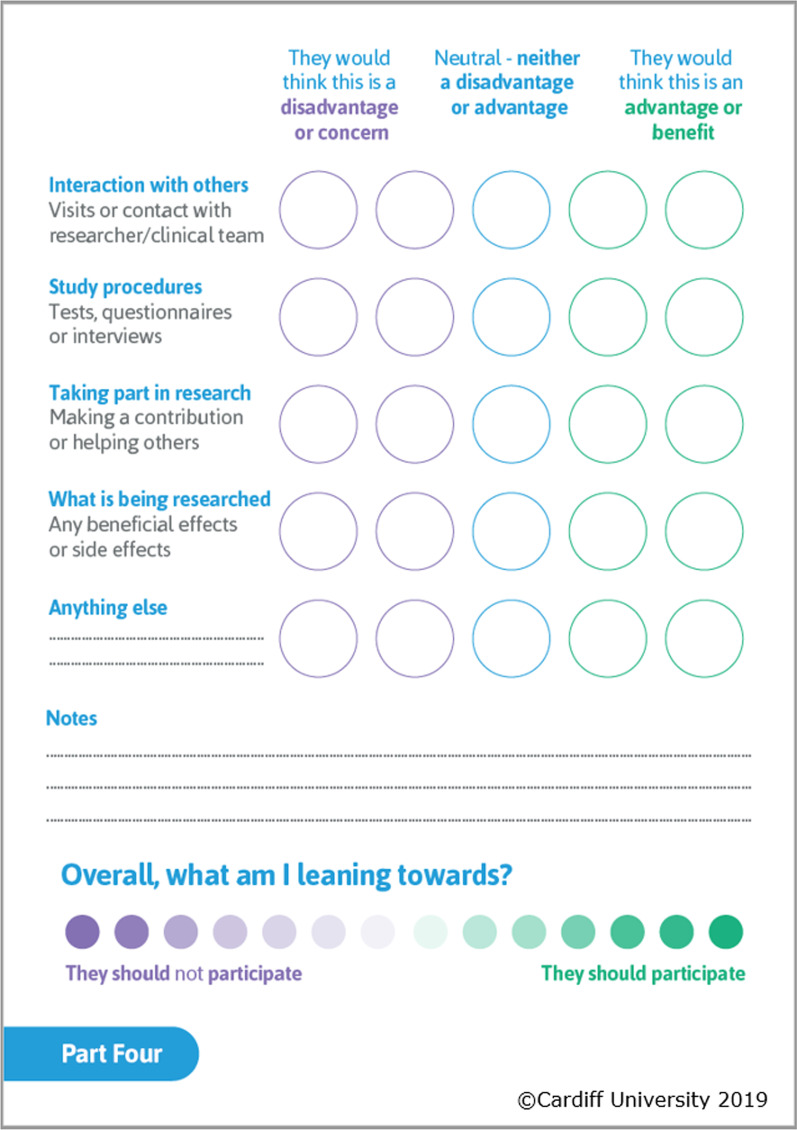
Values clarification exercise included in decision aid

This was then followed by an exercise which used values clarification methods (VCM) [36] to enable proxies to trade-off positive and negative features of the decision in order to facilitate decision-making that was personal and meaningful for the person being represented. As described previously, VCMs are commonly used in DAs and, given the challenges reported by proxies around establishing the research preferences of the person they were representing [9], the decision was made to include an explicit values clarification exercise in this DA. The original choice of visual metaphor for the VCM was a weighing scale, in line with existing DAs for clinical trials [20, 24]. However, the VCM graphic was the area of the DA that most divided the opinions of the lay advisory group, where some members found the visual metaphor as the most valuable part of the DA and others strongly disliked the format. As there is a diverse array of VCMs in use, and the effectiveness of any given VCM is unknown [61], the metaphor was changed to an image that represented both direction and strength of response (Fig. 6).

#### Phase 5: assessing acceptability and comprehensibility of the decision aid

In order to establish the acceptability of the decision support tool, a stakeholder group was convened in phase 5 of the development process. The stakeholder group was an opportunity to consult both those who represent the population who would receive the intervention and those who would be implementers. Nine participants attended a discussion and feedback event which was led with two co-facilitators. Six participants were researchers or research nurses in a range of fields including dementia, stroke, and Multiple Sclerosis, two had experience of caring for a family member living with dementia, and one considered themselves to have prior experience as both a researcher and family carer.

A short acceptability questionnaire adapted from a previous developed acceptability tool [62] was completed by participants, and facilitated small discussion groups enabled participants’ views to be explored in greater detail. The acceptability questionnaire showed that almost all participants felt that the decision support tool was the right length (89%, 8/9) and contained the right amount of information (78%, 7/9), and all participants thought that it would be useful. Open-text responses showed that participants liked the simplicity of the language used and the presentation and ‘flow’ of the information. The colour-coding of sections was thought to be particularly helpful, although different colour combinations were suggested that participants thought would increase the contrast and therefore the readability of the text. The prototype DA was subsequently refined following this feedback.

The discussion groups revealed that participants viewed the format of the decision support tool positively, and participants were universally in favour of it being used within the context of a ‘consultation’ between the researcher and the family member(s) and as a supplement to the Participant Information Sheet. It was thought to be both applicable and useful in a wide range of contexts beyond families of people living with dementia, including in emergency or critical care settings requiring deferred consent or a waiver where family members are approached once the emergency has passed to decide about the continued participation of a patient who lacks capacity. Participants felt that the information contained in the tool was well balanced about participation or non-participation and the potential advantages and disadvantages, and was viewed as empowering families to make an informed decision. Participants recognised that the extra time burden or information burden for family members could act as a barrier but felt that this was offset with the importance of making informed decisions about the inclusion of those who lack capacity.

Further research to explore the feasibility and effectiveness of the intervention is required. However, as this is the first decision support intervention aimed at enhancing proxy decisions about research, establishing which outcomes matter, to whom, and why has not previously been explored. Due to the differences in ‘self/other’ decision-making, outcomes for proxy decisions about trial participation may differ markedly from those reported in trials of interventions to improve informed consent for decisions about trial participation. Therefore, establishing what conceptually constitutes ‘good’ proxy decisions about research, and the generation of a core outcome set, will support the development and subsequent evaluation of interventions. Work to establish a core outcome set for interventions to enhance proxy decisions about research on behalf of adults who lack capacity to consent is underway (COnSiDER registered with Core Outcome Measures in Effectiveness Trials (COMET) Initiative www.comet-initiative.org/studies/details/1409). Candidate measures may include outcomes such as preparedness for decision-making, values-choice congruence, and decision conflict and regret.

## Discussion

Following a growing focus on interventions to improve informed consent for research, the need for interventions to improve proxy decisions about research has now been identified. This paper reports the development of the first such intervention. Informed by the decision-aid literature, theoretical frameworks, intervention development frameworks, and our previous research exploring proxy decision-making for research, we have developed a new decision support intervention for families acting as proxy. Although the intervention (a DA plus an additional education component for the clinician/researcher undertaking recruitment) was primarily informed by research with families of people living with dementia, stakeholders indicated that it is appropriate for use by proxies of people with other conditions associated with cognitive impairment.

## Conclusions

The decision aid is acceptable to both those who would be delivering and receiving the intervention, however formal feasibility testing is needed prior to an evaluation of its effectiveness. As it is a novel intervention, further work to develop and establish appropriate outcome domains and outcome measures is first required.

## Supplementary Information

**Additional file 1**. Logic model for proxy decision support intervention.

## Data Availability

The datasets used during the current study are available from the corresponding author on reasonable request.

## Abbreviations

MCA: Mental Capacity Act 2005
CTR: Medicines for Human Use (Clinical Trials) Regulations SI 2004/1031
CTIMPs: Clinical trials of investigational medicinal products
DA: Decision aid
IPDAS: International Patient Decision Aids Standards
MRC Framework: Medical Research Council Framework for developing and evaluating complex interventions
ODSF: Ottawa Decisional Support Framework
SEM: Socio-ecological model
VCM: Values clarification methods
