# Supplementary material for: Development of a decision support intervention for family members of adults who lack capacity to consent to trials
Source: BMC Med Inform Decis Mak. 2021 Jan 28;21:30. doi: 10.1186/s12911-021-01390-4 (PMC7842028; doi:10.1186/s12911-021-01390-4)
Supplement: Supplementary file 1 — Additional file 1. Logic model for proxy decision support intervention. [file 12911_2021_1390_MOESM1_ESM.docx]

Guide/booklet for families of people living with impaired capacity ‘**Making decisions about research**’ includes:

- general information about inclusion of adults lacking capacity (ALC) in research

- decisions about research (wishes, preferences and values, role of consultee/legal rep)

- relevance of risks and benefits of the study to the person represented

- changing your mind, taking time to decide

- involvement of ALC

Family members understand why people with impaired capacity might be included in research

Family members understand the potential benefits and risks of participation for the person represented

Family members understand the basis for proxy decisions for research (based on wishes and preferences)

Appropriate inclusion of adults with impaired capacity in research

HCPs/researchers confident and competent to include ALC in research

Family members make an informed, considered, authentic decision

Family members of people with impaired capacity who are being approached (or are considering acting) as research proxy use the guide to facilitate discussion and support decision-making during a consultation

HCPs/researchers understand how ALC included in research

HCPs/researchers understand basis for proxy decisions

External and contextual factors

HCPs/researchers who identify and recruit ALC receive online or face-to-face training

Training for health and social care professionals (HCPs) and researchers about adults lacking capacity and research
